# Predicting Future Development of Stress-Induced Anhedonia From Cortical Dynamics and Facial Expression

**DOI:** 10.21203/rs.3.rs-5537951/v1

**Published:** 2025-03-20

**Authors:** Austin A. Coley, Kanha Batra, Jeremy M. Delahanty, Laurel R. Keyes, Rachelle Pamintuan, Assaf Ramot, Jim Hagemann, Christopher R. Lee, Vivian Liu, Harini Adivikolanu, Jianna Cressy, Caroline Jia, Francesca Massa, Deryn LeDuke, Moumen Gabir, Bra’a Durubeh, Lexe Linderhof, Reesha Patel, Romy Wichmann, Hao Li, Kyle B. Fischer, Talmo Pereira, Kay M. Tye

**Affiliations:** 1 The Salk Institute for Biological Studies, La Jolla, CA; 2 University of California, Los Angeles, Los Angeles, CA; 3 University of California, San Diego, La Jolla, CA; 4 Neuroscience Graduate Program, University of California, San Diego, La Jolla,; 5 Medical Scientist Training Program, University of California, San Diego, La Jolla,; 6 Howard Hughes Medical Institute, La Jolla, CA; 7 Kavli Institute for the Brain and Mind; 8 Vrije Universiteit, Amsterdam Netherlands; 9 Northwestern University, Chicago, IL

## Abstract

The current state of mental health treatment for individuals diagnosed with major depressive disorder leaves billions of individuals with first-line therapies that are ineffective or burdened with undesirable side effects. One major obstacle is that distinct pathologies may currently be diagnosed as the same disease and prescribed the same treatments. The key to developing antidepressants with ubiquitous efficacy is to first identify a strategy to differentiate between heterogeneous conditions. Major depression is characterized by hallmark features such as anhedonia and a loss of motivation^1,2^, and it has been recognized that even among inbred mice raised under identical housing conditions, we observe heterogeneity in their susceptibility and resilience to stress^3^. Anhedonia, a condition identified in multiple neuropsychiatric disorders, is described as the inability to experience pleasure and is linked to anomalous medial prefrontal cortex (mPFC) activity^4^. The mPFC is responsible for higher order functions^5–8^, such as valence encoding; however, it remains unknown how mPFC valence-specific neuronal population activity is affected during anhedonic conditions. To test this, we implemented the unpredictable chronic mild stress (CMS) protocol^9–11^ in mice and examined hedonic behaviors following stress and ketamine treatment. We used unsupervised clustering to delineate individual variability in hedonic behavior in response to stress. We then performed *in vivo* 2-photon calcium imaging to longitudinally track mPFC valence-specific neuronal population dynamics during a Pavlovian discrimination task. Chronic mild stress mice exhibited a blunted effect in the ratio of mPFC neural population responses to rewards relative to punishments after stress that rebounds following ketamine treatment. Also, a linear classifier revealed that we can decode susceptibility to chronic mild stress based on mPFC valence-encoding properties prior to stress-exposure and behavioral expression of susceptibility. Lastly, we utilized markerless pose tracking computer vision tools to predict whether a mouse would become resilient or susceptible based on facial expressions during a Pavlovian discrimination task. These results indicate that mPFC valence encoding properties and behavior are predictive of anhedonic states. Altogether, these experiments point to the need for increased granularity in the measurement of both behavior and neural activity, as these factors can predict the predisposition to stress-induced anhedonia.

Anhedonia—described as the inability to experience pleasure and hedonic feeling^12,13^—is an underlying condition and core feature observed in both schizophrenia (SCZ), major depressive disorder (MDD)^14^, and bipolar disorder (BD)^15,16^, and is suggested to be linked to anomalous medial prefrontal cortex (mPFC) activity^4^. The mPFC, a higher order cortical region primarily responsible for cognition^5,6^, working memory^7,8^, sociability^17^, and emotional control^18^, is also involved in valence encoding^19^, essential for discerning positive and negative hedonic values^20^. Stress plays a major role in disrupting mPFC processes leading to depressive-phenotypes and is highly responsive to treatment. Ketamine administration shows promise as an antidepressant for treatment-resistant patients and has notable effects on mPFC cortical neurons^21–23^. Indeed, mPFC imaging studies in MDD patients have identified biomarkers that can predict the response to therapy^24,25^. Recently, non-invasive approaches such as facial expression analysis have been utilized to capture the emotional state of a subject^26,27^. This led us to hypothesize that mPFC valence-encoding processes and behavioral features, including facial expression, can predict future stress-induced phenotypes and response to ketamine.

## Anhedonia classification predicts associative learning performance

**Figure 1:**
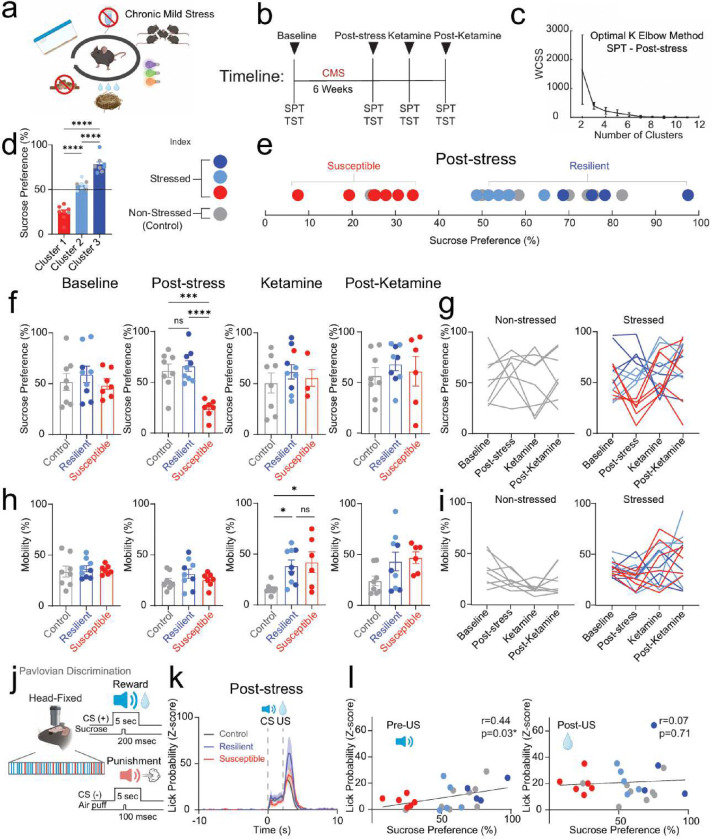
Stress-induced phenotype classification predicts reward task performance **a.** Schematic of unpredictable chronic mild stress (CMS) protocol. CMS mice were exposed to 2–3 stressors per day for 6 weeks that consisted of cage tilting, strobe light illumination, white noise, crowded housing, light/dark cycle manipulations, food deprivation, water deprivation, and damp bedding. **b**. Timeline of measurements for sucrose preference test (SPT) and tail suspension test (TST) during CMS and ketamine treatment. **c.** The optimal k elbow method uses the within-cluster-sum-of-square (WCSS) values to determine the appropriate number of clusters derived from SPT scores of mice at the Post-stress time point. **d.** Cluster analysis of SPT scores for susceptible (cluster 1), neutral (cluster 2), and resilient (cluster 3) groups. Significant decrease in SPT scores from susceptible mice compared to neutral mice (One-way ANOVA, between-subjects F_(2,21)_=100.3, p<0.0001. Tukey Post-hoc, p<0.0001). Significant decrease in SPT scores from neutral mice compared to resilient mice (p<0.0001). Significant increase in SPT scores from resilient mice compared to susceptible mice (p<0.0001). **e.** To determine resilient (dark blue and light blue), and susceptible (red) subjects, k-means clustering (k=3) of sucrose preference scores was applied in both stressed (n=8) and non-stressed control (gray) groups (n=14). **f.** Susceptible mice displayed a reduction in SPT scores compared to control and resilient mice at the Post-stress time point (One-way ANOVA, F_(2,21)_=16.95, p<0.0001, Tukey Post-hoc: control compared to resilient mice, p=0.8051, control compared to susceptible mice, p=0.0003, susceptible compared to resilient mice, p<0.0001 ). No differences were observed at Baseline (One-way ANOVA, F_(2,21)_=0.4606, p=0.6371), Ketamine (One-way ANOVA, F_(2,20)_=0.4637, p=0.6356) or Post-Ketamine time points (One-way ANOVA, F_(2,20)_=0.4364, p=0.6524). **g.** Longitudinal description showing non-stressed control mice (left) and stressed (resilient, neutral, and susceptible) mice during sucrose preference test. **h.** Susceptible and resilient mice displayed an increase in mobility compared to control mice during TST at the Ketamine time point (One-way ANOVA, F_(2,20)_=5.376, p=0.0135, Tukey Post-hoc: control compared to resilient mice, p=0.0309; control compared to susceptible mice, p=0.0246; resilient compared to susceptible mice, p=0.9187. No differences in mobility across groups during Baseline (One-way ANOVA, F_(2,21)_=0.3632, p=0.6997), Post-stress (One-way ANOVA, F_(2,21)_=1.185, p=0.3253), and Post-Ketamine (One-way ANOVA, F_(2,20)_=2.702, p=0.0915) time points. **i.** Longitudinal description showing non-stressed control mice (left) and stressed (resilient, neutral, and susceptible) mice during tail suspension test. **j.** Pavlovian discrimination paradigm in a head-fixed mouse showing US paired with a 5-second pure tone as the conditioned stimulus (CS (+)), with the tone frequency set at 9 kHz for the rewarding CS (sucrose), and a 5-second pure tone as the conditioned stimulus (CS (−)), with the tone frequency set at 2 kHz for the punishment CS (Air Puff). **k.** Peri-stimulus time histogram (PSTH) of lick probability during reward trials in control, resilient, and susceptible mice. **l.** Significant correlation in lick of lick probability and sucrose preference test during CS at Post-stress time point (Pearson’s correlation of lick probability and sucrose preference test in control, resilient, and susceptible mice. left, Pre-US, r=0.44, p=0.03; right, Post-US, r=0.07, p=0.71). Data in bar graphs are shown as mean and error bars around the mean indicate s.e.m. NS, not significant

To test this, we implemented the unpredictable chronic mild stress (CMS) protocol^9–11^ (Fig. 1a) to induce anhedonia and assessed consummatory pleasure, despair, motivation, and sociability across weeks. We used sucrose preference test (SPT) as a measure of anhedonia^9,10^ and utilized unsupervised k-means clustering to classify subjects into resilient and susceptible clusters (Fig. 1b–e). We then evaluated SPT scores in non-stressed (control), resilient and susceptible mice. Our results showed susceptible mice display a significant reduction in sucrose preference following post-stress (Fig. 1f). However, we observed no differences in sucrose preference scores between non-stressed and stressed groups at the baseline, ketamine, and post-ketamine time points (Fig. 1f, g).

**Extended Data Figure 1.**
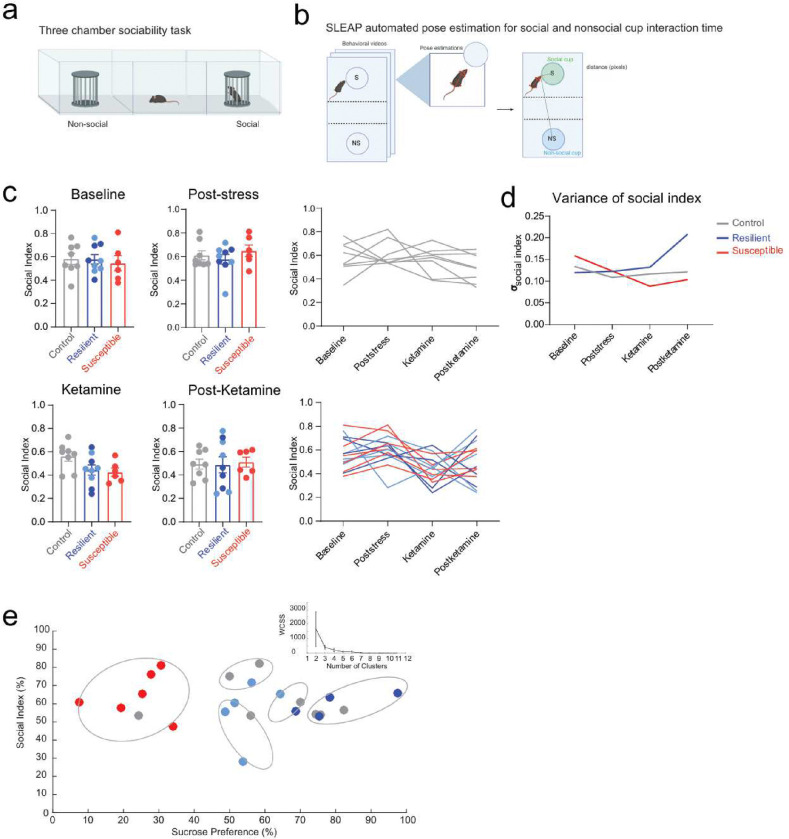
Ketamine treatment after chronic mild stress decreases variance in social index in susceptible mice. **a.** Schematic of three-chamber sociability task assessing social preference. **b.** Workflow for SLEAP automated pose tracking, used to precisely quantify interaction time based on the subject’s distance in pixels and angle to both the social and non-social cups. **c.** No difference in social interaction across groups at Baseline, Post-stress, ketamine, and Post-Ketamine time points. Social index, calculated as a ratio of time spent interacting with the social cup over combined social cup and non-social cup interaction times, measured at Baseline (one-way ANOVA, Tukey’s post hoc, interaction effect: F _(2, 20)_ = 0.1539, p=0.8583), Post-stress (one-way ANOVA, Tukey’s post hoc, interaction effect: F _(2, 20)_=0.09649, p=0.5403), Ketamine (one-way ANOVA, Tukey’s post hoc, interaction effect: F _(2, 20)_=0.2762, p=0.0726), and one week after Ketamine treatment (one-way ANOVA, Tukey’s post hoc, interaction effect: F _(2, 20)_ =3.173, p=0.9614) time points. Error bars represent mean +/− SEM. **d.** Standard deviation plot of social index across Baseline (Control: n=8, SD= 0.1338; Resilient: n=9, SD=0.1196; Susceptible: n=5, SD=0.1585), Post-stress (Control: n=8, SD=0.1087; Resilient: n=9, SD=0.1255; Susceptible: n=5, SD=0.1234), Ketamine (Control: n=8, SD=0.1166; Resilient: n=9, SD=0.1322; Susceptible: n=5, SD=0.08842), and after Ketamine (Control: n=8, SD=0.1213; Resilient: n=9, SD=0.2082; Susceptible: n=5, SD=0.1036) time points. **e.** k-means clustering (k=5) of social index and sucrose preference scores. The optimal k elbow method using the within-cluster-sum-of-square (WCSS) was applied to determine the appropriate number of clusters derived from social index and sucrose preference scores of mice Post-stress time point.

Additionally, CMS mice revealed no difference in mobility during tail suspension test (TST) at baseline or post-stress time points, indicating no difference in behavioral despair or motivation; but showed an increase following ketamine treatment (Fig. 1h, i). These data suggest ketamine application reduces behavioral despair in stressed groups compared to control mice. We observed no significant differences in mobility across groups at the post-ketamine time point. Interestingly, we detected no difference in social preference in susceptible mice in response to CMS (Extended Data Fig. 1).

**Extended Data Figure 2.**
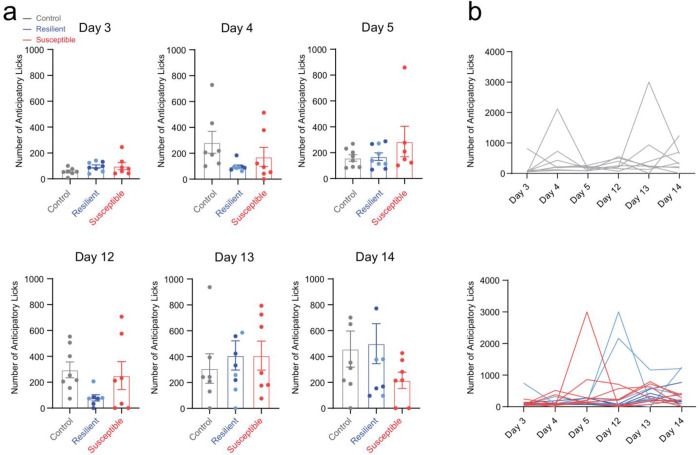
Susceptible mice show no differences in anticipatory licking during head-fixed training task prior to stress. **a.** During head-fixed training, total number of anticipatory licks measured at multiple time points. No significant differences across control, resilient, and susceptible groups: day 3 (one-way ANOVA, Tukey’s post hoc, interaction effect: F _(2, 19)_ =1.644, p=0.2196), day 4 (one-way ANOVA, Tukey’s post hoc, interaction effect: F _(2, 19)_ =2.353, p=0.1221), day 5 (one-way ANOVA, Tukey’s post hoc, interaction effect: F _(2, 20)_ = 1.295, p=0.2958), day 12 (one-way ANOVA, Tukey’s post hoc, interaction effect: F _(2, 19)_ =2.520, p=0.1070), day 13 (one-way ANOVA, Tukey’s post hoc, interaction effect: F _(2, 20)_=0.2470, p=0.7835), and day 14 (one-way ANOVA, Tukey’s post hoc, interaction effect: F _(2, 21)_ = 1.249, p=0.3073) of headfixed training. Error bars represent mean +/− SEM. **b.** Longitudinal description showing non-stressed control mice (top panel: gray) and stressed (bottom panel: resilient and susceptible) mice during headfixed training.

**Extended Data Figure 3.**
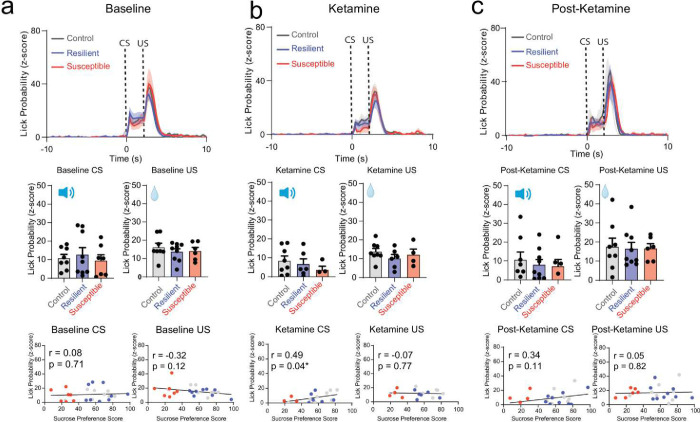
No difference in lick probability within susceptible group at Baseline, Ketamine, and Post-Ketamine time points. **a.** Visualizing lick probability relative to cue onset of CS (0 – 2 seconds) and sucrose delivery of US (2 – 5 seconds) in control, resilient and susceptible groups during Baseline (top panel). No significant differences in lick probability across groups: Lick Probability (One-way ANOVA, Baseline CS, F_(2, 20)_= 0.5011, p= 0.6133; Baseline US, F_(2, 20)_= 0.4939, p= 0.6175) (middle panel). ). No correlation in lick probability and sucrose preference at Baseline. Pearson’s correlation of lick probability and sucrose preference test Baseline CS r= 0.08, p= 0.71, Baseline US r= −0.32, p= 0.12. (bottom panel). **b**. No significant differences in lick probability across groups: Lick probability relative to cue onset of CS and sucrose delivery of US in control, resilient and susceptible groups during Ketamine time point (top panel). Lick Probability (One-way ANOVA, Ketamine CS, F_(2, 15)_= 0.8240, p= 0.4576; Ketamine US, F_(2, 20)_= 0.2545, p=0.7778) (middle panel). Significant correlation in lick probability and sucrose preference at Ketamine time point during CS, but not US. Pearson’s correlation of lick probability and sucrose preference test Ketamine CS r= 0.49, p= 0.039* Ketamine US r= −0.07, p= 0.77 (bottom panel). **c.** No significant differences in lick probability across groups: Lick probability relative to cue onset of CS and sucrose delivery of US in control, resilient and susceptible groups during post-Ketamine timepoint (top panel). Lick Probability (One-way ANOVA, post-Ketamine CS, F_(2, 20)_= 0.0239, p=0.9764. (middle panel). No correlation in lick probability and sucrose preference at post-Ketamine. Pearson’s correlation of lick probability and sucrose preference test post-Ketamine CS r= 0.34, p= 0.11, post-Ketamine US r= 0.05, p= 0.82 (bottom panel).

To assess the impact of chronic stress on the neural and behavioral readouts for reward or punishment-predictive cues, we trained mice that would ultimately undergo CMS or their non-stressed controls in a head-fixed Pavlovian discrimination task used to discriminate reward-predictive and punishment-predictive stimuli (Fig. 1j). During the task, one conditioned stimulus (CS) is paired (tone) with a 30% sucrose solution delivery reward (US-unconditioned stimulus), and a different conditioned stimulus is paired with a punishment air puff. We observed no significant differences in anticipatory licking between stressed groups during the training phase (Extended Data Fig. 2). Our results showed no difference between groups in lick probability in reward trials during the anticipatory phase (following CS onset and prior to US delivery) and consummatory phase (following US delivery) at the post-stress time point (Fig. 1k). Additionally, we measured lick probability during baseline, ketamine, and post-ketamine time points and observed no differences between groups during the CS or US phases (Extended Data Fig. 3). However, we did detect a significant correlation in lick probability and sucrose preference in all mice during the conditioned stimulus at the post-stress time point; suggesting that susceptible mice display both a reduction in lick probability and sucrose preference (Fig. 1l). No detectable correlation was observed during the unconditioned stimulus (Fig. 1l). These findings suggest that anhedonia classification can predict reward consumption performance during post-stress time points.

## Chronic stress blunts mPFC valence population dynamics and recovers at post-ketamine time point

**Extended Data Figure 4.**
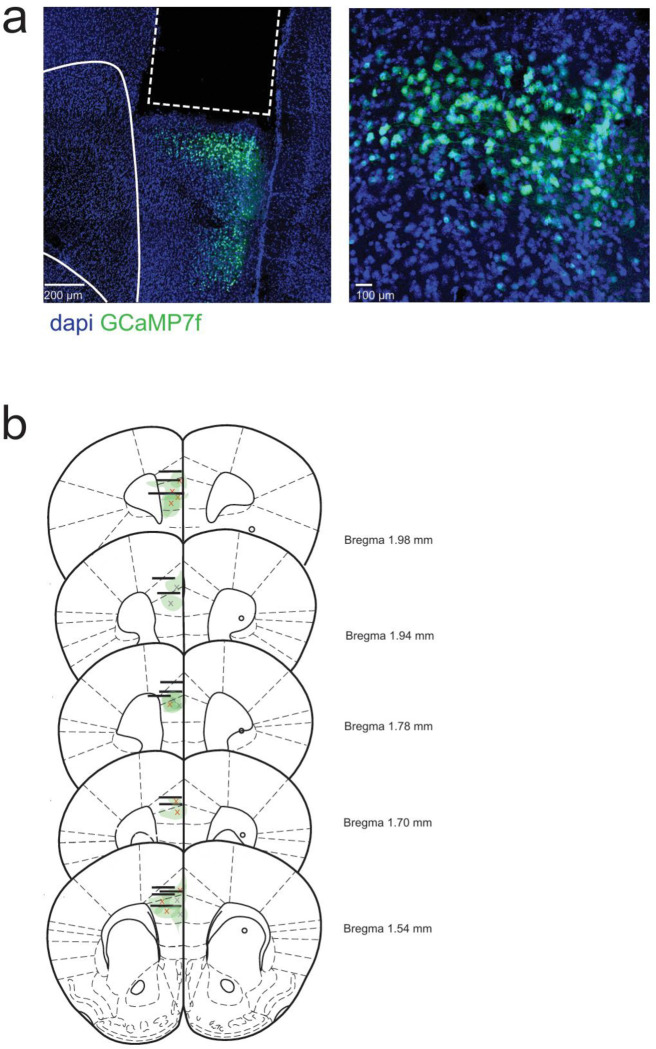
Histological validation of injection sites and implants. **a.** Representative images of GRIN lens implant and GCaMP7f expression in the PFC **b.** GRIN lens implant locations and GCaMP7f injection sites in the mPFC for in vivo 2-photon calcium recording (Bregma 1.54 to 1.98 mm). x indicates viral injection site.

**Extended Data Figure 5.**
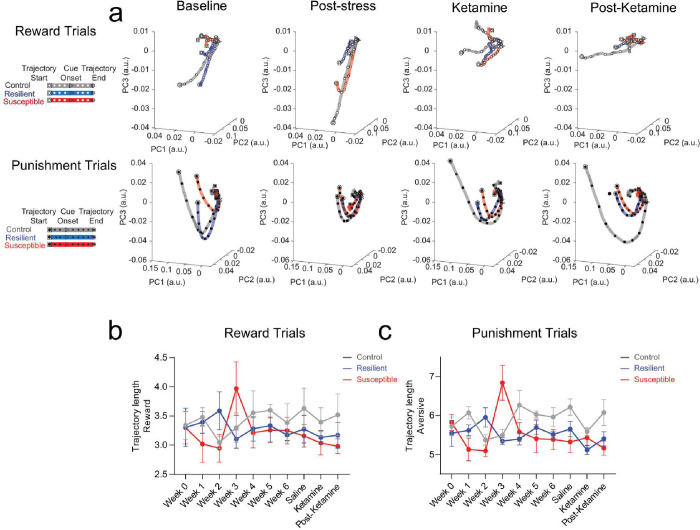
Visualizing neural population activity as neural trajectories using local Z-score revealed no differences across weeks. **a.** Neural trajectory lengths (post-event, 0–10 sec) in control, resilient, and susceptible groups during reward trials and punishment trials (using principal components that captured 90% of variance) across weeks. **b.** Reward (Left panel): Mixed ANOVA: subjects, F_(1.928, 109.9)_=8.184, p=0.0006, weeks, F_(9,114)_=1.638, p=0.1127, interaction, F_(18,114)_=4.126. **c.** Punishment (Right panel): subjects, F_(1.984, 113.1)_=8.475, p=0.0004, weeks, F_(9,114)_=1.154, p=0.3313, p<0.0001, interaction, F_(18,114)_=3.140, p=0.0001.

**Figure 2:**
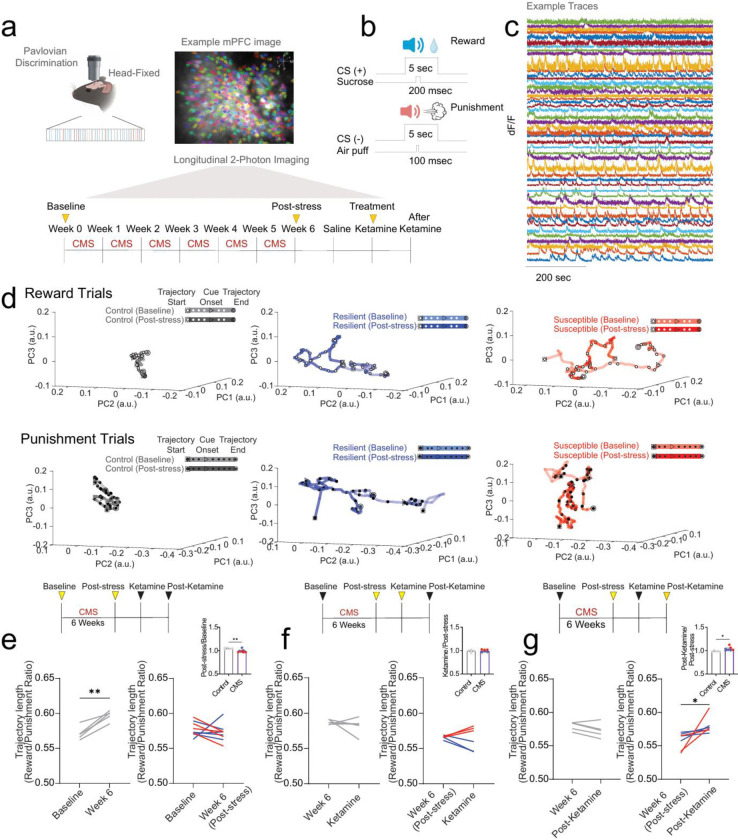
Chronic stress blunts mPFC valence population dynamics ratio while a single dose of ketamine reverses this effect **a.** Head-fixed mouse and example mPFC 2-Photon image highlighting region of interest (ROI) neurons. Experimental paradigm shows the timeline of longitudinal 2-Photon imaging sessions. **b.** Pavlovian discrimination paradigm task showing Sucrose Reward trials (US paired with a 5-second tone (CS+)) and Air puff Punishment trials (US paired with a 5-second tone (CS−)). **c.** Example df/f traces of mPFC neurons. **d.** To explore population dynamics, we applied principal component analysis (PCA) of neural trajectories of ROI matched (co-registered) mPFC neurons during reward trials (Top) and punishment trials (Bottom) showing control (gray), resilient (blue), and susceptible (red) groups in a lower dimensional common principal component (PC) sub-space from Baseline to Post-stress time points. The first PCs capture 42.97% of the variance. The top 23 PCs were used to capture 59.51% of the variance. **e.** To examine the reward and punishment population dynamics we examined we used a super global Z-score (Z-score normalized across multiple sessions) and measured the trajectory lengths (post-event, 0–10 sec) during reward and punishment trials in pairwise (time point matched) ROI matched co-registered neurons and calculated the reward/punishment ratio during baseline to post-stress time points. Control mice showed an increase in reward/punishment ratio over time; Control (left), paired t-test, p=0.0031. Stressed mice showed no difference: CMS (right), paired t-test, p=0.3805. Significant decrease in trajectory length ratio (ratio normalized to baseline time point) in CMS mice compared to control mice. Bar graph: unpaired t-test, p=0.0031. **f.** No significant differences were observed in pairwise ROI matched neural trajectory lengths (post-event, 0–10 sec) reward/punishment ratio during Post-stress to Ketamine time points: Control (left), paired t-test, p=0.4520; CMS (right), paired t-test, p=0.8203. Bar graph: unpaired t-test, p=0.6929 **g.** Stressed groups showed an increase in reward/punishment ratio in pairwise ROI matched neural trajectory lengths (post-event, 0–10 sec) reward/punishment ratio during Post-stress to Post-Ketamine time points CMS (right), paired t-test, p=0.0475. No significant differences were observed in control groups. Control (left), paired t-test, p=0.0774. Significant increase in trajectory length ratio (ratio normalized to Post-stress time point) in CMS mice compared to control mice. Bar graph: unpaired t-test, p=0.0277. *p<0.05, **p<0.01.

To examine the relative dynamics of responses to reward- and punishment-predictive cues, we utilized longitudinal *in vivo* 2-Photon calcium imaging to track mPFC neuronal population activity (Extended Data Fig. 4), while mice are performing a Pavlovian discrimination task across 10 weeks during chronic mild stress and ketamine treatment (Fig. 2a–c). Using a local z-score (normalized to the baseline for each trial), we applied principal component analysis (PCA) to plot activity in a lower dimensional space during reward and punishment trials (Extended Data Fig. 5a). We examined population dynamics across weeks in non-stressed control, resilient and susceptible groups by measuring trajectory length post CS onset (0–10 sec) during reward trials and punishment trials (Extended Data Fig. 5b, c). Longer trajectories reflect more dynamic population activity during the trial^28^. Our results showed no differences across groups during reward trials (Extended Data Fig. 5b). During punishment trials, we observed no differences in trajectory lengths at stress time points (Extended Data Fig. 5c).

**Extended Data Figure 6.**
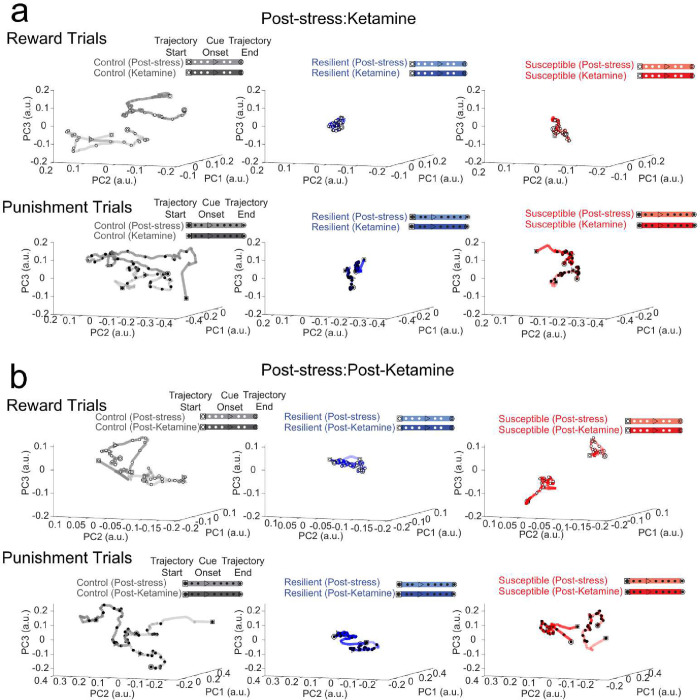
Neural trajectories of longitudinally imaged ensembles during Post-stress to Ketamine time points, and Post-stress to Post-Ketamine time points. **a.** Using neural trajectories of mPFC neural populations plotted with a super global Z-score (Z-score normalized across multiple sessions), ROI-matched populations between sessions during reward (Top) and punishment trials (Bottom) at Post-stress and Ketamine time points. **b.** ROI matched neural trajectories of mPFC neural populations during reward (Top) and punishment trials (Bottom) at Post-stress and Post-Ketamine time points.

To further evaluate the evolution of responses to reward- and punishment-predictive cues in mPFC neurons, we tracked and matched individual single cells over weeks and calculated the PCA trajectory length reward/punishment ratio in response to chronic stress and ketamine treatment as a reflection of the relative change in population dynamics (Fig. 2d; Extended Data Fig. 6a, b). Our results showed an increase in the reward/punishment ratio from baseline to week 6 (post-stress time point) in control mice, indicating an increase in mPFC reward processing over time (Fig. 2e). Subjects exposed to chronic mild stress displayed no difference in population dynamics ratio from baseline to post-stress (Fig. 2e). We then measured the reward/punishment balance from post-stress to ketamine periods, and observed no difference in control or stressed groups (Fig. 2f, Extended Data Fig. 6a). Interestingly, when examining the difference from post-stress to post-ketamine time points we revealed an increase in mPFC trajectory length reward/punishment ratio in stress subjects (Fig. 2g, Extended Data Fig. 6b), indicating an increase in reward processing preference in both resilient and susceptible groups one week following ketamine treatment. We observed no difference in reward/punishment balance in control mice at post-stress to post-ketamine periods, suggesting stress-dependent changes in response to ketamine (Extended Data Fig. 6b).

## mPFC population activity predicts anhedonia phenotypes prior to stress exposure

**Extended Data Figure 7.**
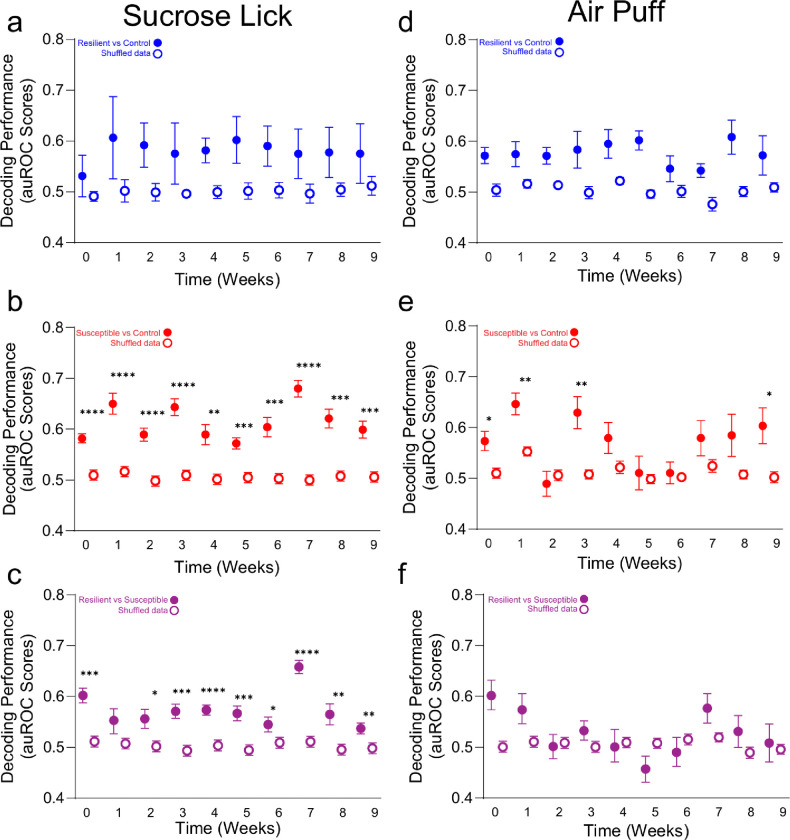
mPFC population activity decodes stress phenotypes. **a.** Significant decoding accuracy of *resilient vs control groups* compared to shuffled data. Decoding accuracy in response to sucrose lick in *resilient* vs *control* groups across weeks. Two-way Repeated Measures ANOVA, event F_(1,18)_=143.5, p<0.0001, weeks F_(3.899,70.17)_=2.145, p=0.0858, interaction F_(9,162)_=1.309, p=0.2361. **b.** Significant decoding accuracy of *susceptible vs control* groups compared to shuffled data within individual weeks. Decoding accuracy in response to sucrose lick in susceptible vs *control* groups across weeks. Two-way Repeated Measures ANOVA, event F_(1,18)_=197.2, p<0.0001, weeks F_(3.788,68.18)_=4.813, p=0.0021, interaction F_(9,162)_=4.230, p<0.0001. Tukey Post-hoc, Weeks 0–9: p<0.0001, p<0.0001, p<0.0001, p<0.0001, p=0.0014, p=0.0001, p=0.0004, p<0.0001, p=0.0001, p=0.0003. **c.** Significant decoding accuracy of *resilient* vs *susceptible groups* compared to shuffled data within individual weeks. Decoding accuracy in response to sucrose lick in *resilient* vs *susceptible* groups across weeks. Two-way Repeated Measures ANOVA, event F_(1,18)_=234.8, p<0.0001, weeks F_(4.550,81.91)_=5.171, p=0.0005, interaction F_(9,162)_=3.633, p=0.0004. Tukey Post-hoc, Weeks 0–9: p=0.0001, p=0.1186, p=0.0171, p=0.0003, p<0.0001, p=0.0007, p=0.0384, p<0.0001, p=0.0081, p=0.0055. **d.** No significant difference in decoding accuracy of *resilient vs control groups* compared to shuffled data across weeks. Decoding accuracy in response to Air puff in *resilient* vs *control* groups across weeks. Two-way Repeated Measures ANOVA, event F_(1,18)_=72.28, p<0.0001, weeks F_(4.292,77.26)_=1.041, p=0.3943, interaction F_(9,162)_=0.5241, p=0.8556. **e.** Significant decoding accuracy of *susceptible vs control groups* compared to shuffled data within individual weeks, but not week 2, and weeks 4–8. Decoding accuracy in response to Air puff in susceptible vs *control* groups across weeks. Two-way ANOVA, event F_(1,18)_=51.47, p<0.0001, weeks F_(5.353,96.35)_=3.086, p=0.0028, interaction F_(9,162)_=1.883, p=0.0579. Tukey Post-hoc, Weeks 0–9: p=0.0105, p=0.0017, p=0.5491, p=0.0036, p=0.1050, p=0.7347, p=0.7196, p=0.1682. **f.** No significant difference in decoding accuracy of *resilient vs susceptible groups* across weeks. Decoding accuracy in response to Air puff in *resilient* vs *susceptible* groups across weeks. Two-way Repeated Measures ANOVA, event F_(1,18)_=7.780, p=0.0121, weeks F_(4.555,81.99)_=2.203, p=0.0676, interaction F_(9,162)_=2.225, p=0.0229. All post-hoc comparisons are Tukey t-tests, *p<0.05, **p<0.01, ***p<0.001, ****p<0.0001 All 2-way ANOVAs were for event (event vs shuffle) and weeks (0–9).

**Figure 3:**
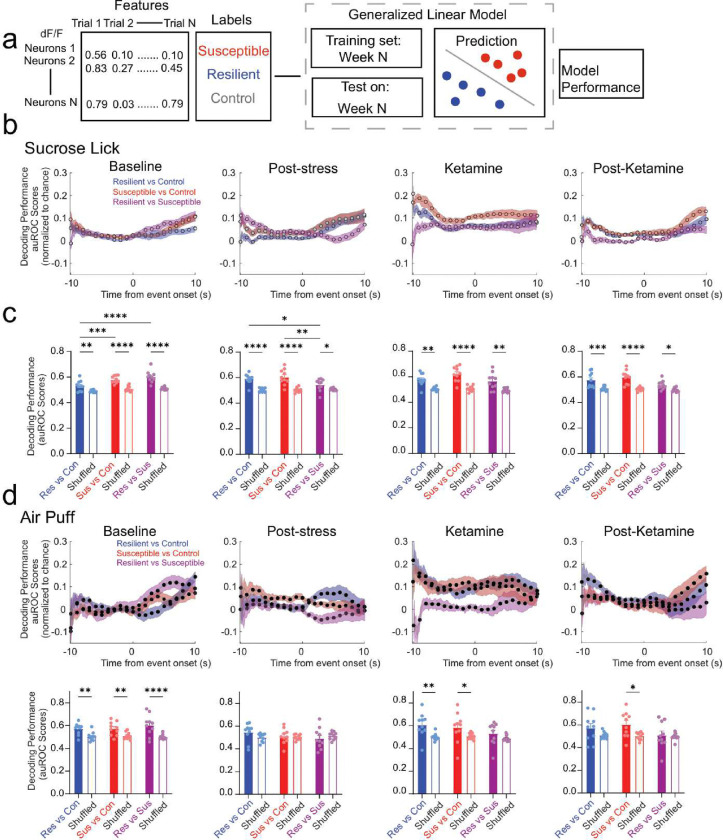
mPFC population dynamics predicts future resilience or susceptibility, before stress exposure **a**. Schematic depicts feature and label inputs for the generalized linear model classifier used for decoding performance. **b.** Decoding performance across time during the first sucrose lick following US presentation (reward trials) in *resilient* vs *control* groups (blue), *susceptible* vs *control* groups (red), and *resilient vs*. *susceptible* groups (purple) at Baseline, Post-stress, Ketamine, and Post-Ketamine time points. **c.** Decoding accuracy during Sucrose lick (first lick following sucrose presentation). *Susceptible* vs *control* groups displayed a significantly greater decoding performance than *resilient* vs *control* groups at Baseline (Two-way ANOVA, event F_(1,27)_=86.98, p<0.0001, groups F_(2,27)_=11.91, p=0.0002, interaction, F_(2,27)_=4.175, p=0.0263. Tukey Post-hoc, *resilient* vs *control* compared to *susceptible* vs *control* groups, p=0.0010, *resilient* vs *control* compared *resilient vs*. *susceptible* groups. p<0.0001. Significantly greater decoding accuracy in *resilient* vs *control* compared to *resilient vs*. *susceptible* groups, and *susceptible* vs *control* groups compared to *resilient vs*. *susceptible* groups at the Post-stress time point (Two-way ANOVA, event F_(1,27)_=58.08, p<0.0001, groups F_(2,27)_=3.0009, p=0.0661, interaction, F_(2,27)_=4.110, p=0.0277. Tukey Post-hoc, *resilient* vs *control* compared to *resilient vs*. *susceptible* groups, p=0.0216, *susceptible* vs *control* groups compared to *resilient vs*. *susceptible* groups, p=0.0019. Stress phenotypes displayed a significantly higher decoding performance compared to shuffled data at each time point, but no differences were observed across groups at Ketamine (Two-way ANOVA, event F_(1,27)_=203.4, p<0.0001, groups F_(2,27)_=3.693, p=0.0382, interaction, F_(2,27)_=1.450, p=0.2522) and Post-Ketamine time points (Two-way ANOVA, event F_(1,27)_=55.42, p<0.0001, groups F_(2,27)_=4.134, p=0.0272, interaction, F_(2,27)_=3.203, p=0.0564). **d.** Time series traces depicting decoding performance during air puff-US (punishment trials) in *resilient* vs *control* groups (blue), *susceptible* vs *control* groups (red), and *resilient* vs *susceptible* groups (purple) at Baseline, Post-stress, Ketamine, and Post-Ketamine time points. **e.** Decoding accuracy during Air puff-US. Significantly greater decoding performance of *resilient* vs *control* groups and *Susceptible* vs *control* groups compared to shuffled data at Baseline (Two-way ANOVA, event F_(1,27)_=41.80, p<0.0001, groups F_(2,27)_=0.2737, p=0.7627, interaction, F_(2,27)_=1.056, p=0.3617), and the Ketamine time points (Two-way ANOVA, event F_(1,27)_=14.46, p=0.0007, groups F_(2,27)_=1.437, p=0.2552, interaction, F_(2,27)_=0.9261, p=0.4083), but no differences across stress groups. No difference in decoding performance of *resilient* vs *control* groups to shuffled data and *Susceptible* vs *control* groups compared to shuffled data at the Post-stress time point (Two-way ANOVA, event F_(1,27)_=0.3822, p=0.5416, groups F_(2,27)_=0.6345, p=0.5379, interaction, F_(2,27)_=1.679, p=0.2054). Mice with a susceptible phenotype displayed a significantly greater decoding performance compared to shuffled data at Post-Ketamine time point, but no differences were observed across stress groups (Two-way ANOVA, event F_(1,27)_=65.09, p<0.0001, groups F_(2,27)_=1.840, p=0.1782, interaction, F_(2,27)_=1.392, p=0.2659). All post-hoc comparisons are Tukey t-tests, *p<0.05, **p<0.01, ***p<0.001, ****p<0.0001 All 2-way ANOVAs were for event (event vs shuffle) and groups (*resilient* vs *control*, *susceptible* vs *control,* and *resilient* vs *susceptible*). Data in bar graphs are shown as mean and error bars around the mean indicate s.e.m.

To determine if mPFC population activity encodes stress-induced anhedonia behavioral phenotype classification, we utilized a generalized linear model (GLM) to predict if mPFC neuronal population activity could decode control, resilient and susceptible groups (Fig. 3a). We trained and tested neural data acquired from the first sucrose lick during reward trials and air puff during punishment-US across weeks, and analyzed decoding performance for *resilient* vs *control* groups, *susceptible* vs *control* groups, and *resilient* vs *susceptible* groups. Our results showed there is a high decoding accuracy for *resilient* vs *control* groups compared to shuffled data during first sucrose lick during individual weeks (Extended Data Fig. 7a). In *susceptible* vs *control* groups, we observed a significantly greater decoding performance during sucrose lick at all time points; and most weeks were distinguishable for *resilient* vs *susceptible* performance with the exception of week 1 (Extended Data Fig. 7b, c.). These data suggest that mPFC population activity can be used to discern susceptible and resilient phenotypes in response to first sucrose lick.

We then compared decoding accuracy between stress groups at baseline, post-stress, ketamine, and post-ketamine time points during first sucrose lick. Interestingly, at baseline, we observed a significant increase in decoding performance in susceptible vs *control* groups compared to *resilient* vs *control* groups (Fig. 3b, c). These data suggest that mPFC neural population activity in susceptible mice is more distinct compared to resilient mice in response to reward stimuli prior to stress. Additionally, at the post-stress, ketamine, and post-ketamine time points, we observed a significantly greater decoding performance in both *susceptible* vs *control* and *resilient* vs *control* groups. These data indicate mPFC population activity can decode anhedonia phenotypes during stress and ketamine treatment in response to first sucrose lick.

Next, we examined decoding performance in response to air puff between *resilient* vs *control* groups, *susceptible* vs *control* groups, and *resilient* vs *susceptible* groups across weeks (Extended Data Fig. 7d–f). The *susceptible* vs *control* groups displayed a significant increase in decoding accuracy compared to shuffle data within individual weeks except at an early stress time point (week 2), and late stress time points (weeks 4–8) (Extended Data Fig. 7e). Interestingly, we observed no significant differences in decoding accuracy across weeks in *resilient* vs *control* groups or *resilient vs*. *susceptible* groups in response to air puff stimuli (Extended Data Fig. 7d, f). These data suggest that *susceptible* vs *control* groups displayed distinct mPFC activity encoding properties in response to air puff during stress.

To measure the difference in *resilient* vs *control*, *susceptible* vs *control*, and *resilient* vs *susceptible* groups in response to air puff stimuli we measured the decoding accuracy at baseline, post-stress, ketamine, and post-ketamine time points (Fig. 3d). At baseline, we were able to significantly decode resilient mice from control mice, susceptible from control, and resilient from susceptible groups compared to shuffled data (Fig. 3e). But we did not detect a difference amongst the *resilient* vs *control* compared *to susceptible* vs *control* at baseline (Fig. 3e). During post-stress, the *resilient* vs *control*, *susceptible* vs *control*, and *resilient* vs *susceptible* groups displayed no difference compared to shuffled data (Fig. 3e). These data demonstrate chronic mild stress ablates phenotype decoding accuracy during punishment trials.

## Facial expression features decode stress phenotypes

**Figure 4:**
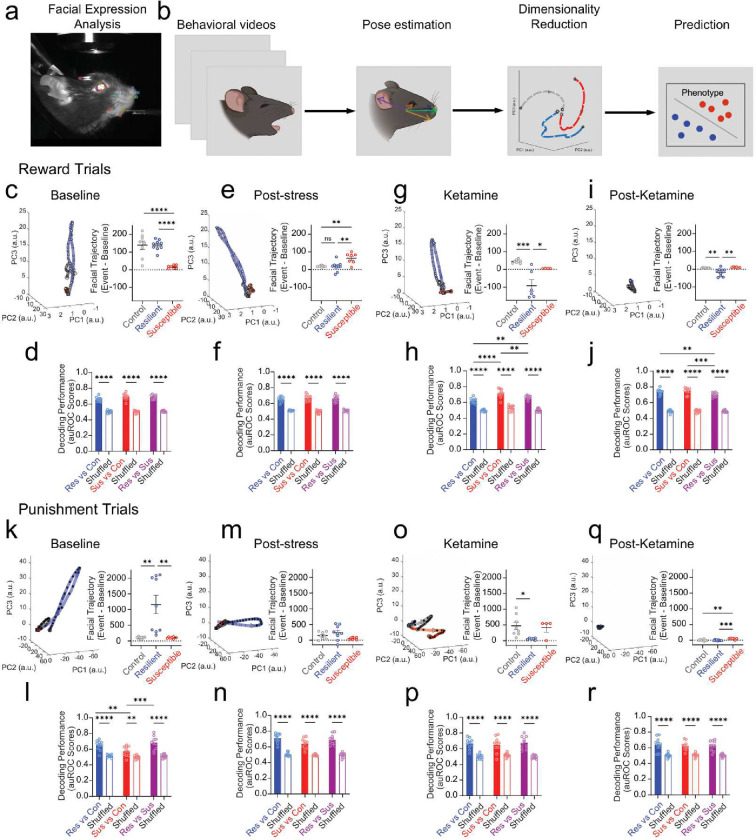
Susceptibility and Resilience can be decoded and predicted from facial expression dynamics **a.** Example image of labeled mouse facial features. **b.** To determine if we could predict future responses to stress or responses to ketamine based on facial features alone, we first extracted facial keypoints from using SLEAP, then plotted the facial expression dynamics in a dimensionality-reduced trajectory in time across principal component space of facial expression dynamics. **c.** To measure facial dynamics, we used a local Z-score, and extracted PCA trajectories (top 3 PCs capture 81.91% of the variance; 8 PCs were used to capture 90.54% of the variance) of facial features at baseline (left) and difference score (right) of trajectory lengths post-event (10 sec CS) – pre-event (10 sec Pre-CS). Control and resilient groups displayed a significantly greater PCA difference score compared to susceptible mice. One-way ANOVA, between-subjects F_(2,21)_=20.18, p<0.0001. Tukey Post-hoc, Control compared to Resilient mice, p=0.9994, Control compared to Susceptible mice, p<0.0001, Resilient compared to Susceptible mice, p<0.0001) **d.** Significantly greater facial decoding accuracy in stressed groups compared to shuffled data, but no difference across stressed groups during reward trials at Baseline (Two-way ANOVA, event F_(1,18)_=573.2, p<0.0001, groups F_(2,36)_=3.095, p=0.0575, interaction, F_(2,36)_=3.206, p=0.0523). **e.** Susceptible groups displayed a significant increase in PCA difference score compared to control and resilient groups at Post-stress (One-way ANOVA, F_(2,21)_=9.139, p=0.0014. Tukey Post-hoc, Control compared to Resilient mice, p=0.9784, Control compared to Susceptible mice, p=0.0045, Resilient compared to Susceptible mice, p=0.0023). **f.** Significantly greater facial decoding accuracy in stressed groups compared to shuffled data, but no difference across groups at Post-stress time point. (Two-way ANOVA, event F_(1,18)_=344.3, p<0.0001, groups F_(2,36)_=0.1186, p=0.8885, interaction, F_(2,36)_=1.898, p=0.1645). **g.** Resilient mice displayed a significant reduction in PCA difference score compared to control and susceptible groups at Ketamine time point (One-way ANOVA, F_(2,15)_=15.18, p=0.0002. Tukey Post-hoc, Control compared to Resilient mice, p=0.0002, Control compared to Susceptible mice, p=0.3646, Resilient compared to Susceptible mice, p=0.0143). **h.** Significantly greater decoding accuracy in stressed groups compared to shuffled data, and a significantly greater increase in *susceptible vs control* groups compared to *resilient vs control* groups at Ketamine time point (Two-way ANOVA, event F_(1,18)_=255.9, p<0.0001, groups F_(2,36)_=21.30, p<0.0001, interaction, F_(2,36)_=5.525, p=0.0081. Tukey Post-hoc, *control vs resilient* compared to *Control vs Susceptible* groups, p<0.0001, *Control vs Resilient* groups compared to *Resilient vs Susceptible* groups, p=0.0068, *Control vs Susceptible* groups compared to *Resilient vs Susceptible* groups, p=0.0023. **i.** Resilient mice displayed a significant reduction in PCA difference score compared to control and susceptible groups at Post-Ketamine time point (One-way ANOVA, F_(2,20)_=9.206, p=0.0015. Tukey Post-hoc, Control compared to Resilient mice, p=0.0054, Control compared to Susceptible mice, p=0.9070, Resilient compared to Susceptible mice, p=0.0038. **j.** We found a significantly greater decoding accuracy in stressed groups compared to shuffled data, and a significantly higher decoding accuracy in *resilient vs control* groups compared to *resilient vs susceptible* groups and *Susceptible vs Control* compared to *Resilient vs Susceptible* groups at the Post-Ketamine time point (Two-way ANOVA, event F_(1,18)_=665.3, p<0.0001, groups F_(2,36)_=6.825, p=0.0031, interaction, F_(2,36)_=5.316, p=0.0095. Tukey Post-hoc, *resilient vs control* compared to *susceptible vs control*, p=0.6321, *resilient vs control* groups compared to *resilient vs susceptible* groups, p=0.0019, *susceptible vs control* groups compared to *resilient vs susceptible* groups, p=0.0001). **k.** Resilient groups displayed a significant increase in PCA difference score compared to control and susceptible groups at Baseline during punishment trials (One-way ANOVA, F_(2,21)_=10.85, p=0.0006. Tukey Post-hoc, Control compared to Resilient mice, p=0.0016, Control compared to Susceptible mice, p>0.9999, Resilient compared to Susceptible mice, p=0.0023). **l.** We observed a significantly greater decoding accuracy in stressed groups compared to shuffled data, and a significantly greater increase in *resilient vs control* compared to *susceptible vs control* groups at Baseline (Two-way ANOVA, event F_(1,18)_=91.33, p<0.0001, groups F_(2,36)_=7.033, p=0.0026, interaction, F_(2,36)_=4.068, p=0.0255. Tukey Post-hoc, *resilient vs control* groups compared to *susceptible vs control* groups, p=0.0077, *resilient vs control* groups compared to *resilient vs susceptible* groups, p=0.3890, *susceptible vs control* compared to *resilient vs susceptible* groups, p=0.0002). **m.** No differences in PCA difference scores at the Post-stress time point (One-way ANOVA, F_(2,21)_=2.884, p=0.0782). **n.** Significantly greater decoding accuracy in stressed groups compared to shuffled data, but no difference across groups at the Post-stress time point (Two-way ANOVA, event F_(1,18)_=230.7, p<0.0001, groups F_(2,36)_=3.343, p=0.0466, interaction, F_(2,36)_=2.133, p=0.1357). **o.** Resilient mice displayed a significant reduction in PCA difference score compared to control and susceptible groups at Ketamine time point (One-way ANOVA, F_(2,15)_=4.651, p=0.0268. Tukey Post-hoc, Control compared to Resilient mice, p=0.0256, Control compared to Susceptible mice, p=0.9246, Resilient compared to Susceptible mice, p=0.1224). **p.** Significantly greater decoding accuracy in stressed groups compared to shuffled data at Ketamine, but no difference across groups at the Ketamine time point (Two-way ANOVA, event F_(1,18)_=70.99, p<0.0001, groups F_(2,36)_=0.02305, p=0.9772, interaction, F_(2,36)_=1.060, p=0.3571). **q.** Susceptible mice displayed a significant increase in PCA difference score compared to control and resilient groups (One-way ANOVA, F_(2,20)_=12.58, p=0.0003. Tukey Post-hoc, Control compared to Resilient mice, p=0.6814, Control compared to Susceptible mice, p=0.0022, Resilient compared to Susceptible mice, p=0.0003. **r.** Significantly greater decoding accuracy in stressed groups compared to shuffled data at Post-Ketamine, but no difference across groups (Two-way ANOVA, event F_(1,18)_=56.50, p<0.0001, groups F_(2,36)_=0.2553, p=0.7915, interaction, F_(2,36)_=0.3098, p=0.7355). Data in bar graphs are shown as mean and error bars around the mean indicate s.e.m.

To further evaluate the affective state of subjects exposed to chronic stress, we utilized markerless pose tracking system SLEAP to examine the facial features in response to reward and punishment trials (Fig. 4a). To capture the spatiotemporal dynamics of the coordination of facial features, we extracted high dimensional facial data from videos and then plotted this in reduced dimensional space using principal component analysis to track facial expression dynamics in response to stress and ketamine treatment (Fig. 4b). Similar to neural analysis, using a local-z-score, we examined facial dynamics prior to and across stress exposure in control, resilient and susceptible groups by measuring facial trajectory length difference score (Post-event - baseline) during reward trials (Supplementary Video 1). At baseline, our results show reduced facial trajectory lengths difference score in susceptible mice compared to control and resilient groups (Fig. 4c). We observed opposing results at post-stress, where susceptible mice displayed an increase in trajectory lengths difference score during reward trials (Fig. 4e). Following ketamine administration, susceptible mice showed an increase in facial dynamics compared to resilient mice (Fig. 4g). Similarity, at the post-ketamine time point, susceptible mice displayed a significant increase trajectory lengths difference score compared to resilient mice (Fig. 4i).

**Extended Data Figure 8.**
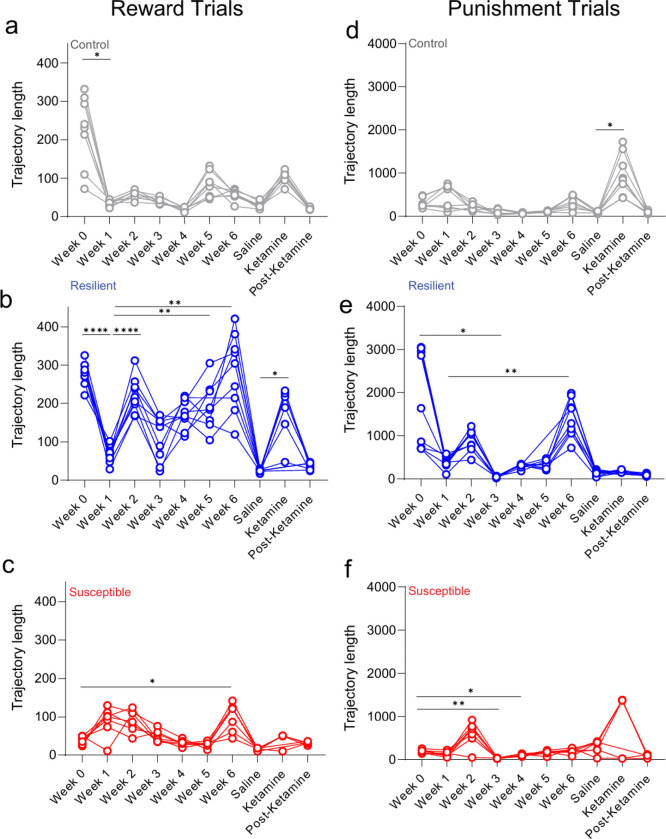
Resilient mice display increase in facial feature dynamics during chronic stress **a.** Significant reduction in PCA trajectory lengths from week 0 to week 1 in control mice. Trajectory lengths post-event (0–10 sec at on-set of CS) during reward trials. Mixed ANOVA control mice (top): weeks, F_(1.367, 10.64)_=29.06, p=0.0001. Tukey Post-hoc, p=0.0114 **b**. Significant reduction in PCA trajectory lengths from week 0 to week 1, and increases in week 2, 5, 6, and Ketamine weeks in resilient mice. resilient groups (middle): weeks, F_(2.777, 20.98)_=32.17, p<0.0001. Tukey Post-hoc, Weeks: 0/1, p<0.0001, 1/5, p=0.0063, 1/6, p=0.0031 Saline/Ketamine, p=0.0305. **c.** Significant increase in PCA trajectory lengths at week 6 in susceptible mice (bottom): weeks, F_(2.030, 12.63)_=13.43, p=0.0007. Tukey Post-hoc, Weeks: 0/6, p=0.0212. **d.** Significant increase in PCA trajectory lengths at Ketamine in control mice. Trajectory lengths post-event (0–10 sec at on-set of CS) during punishment trials. Mixed ANOVA control mice (top): weeks, F_(1.544, 12.08)_=17.28, p=0.0005. Tukey Post-hoc, Saline/Ketamine, p=0.0171. **e.** Significant reduction in PCA trajectory lengths from week 0 to week 3, accompanied with an increase from week 1 to week 6 in resilient mice. resilient mice (middle): weeks, F_(1.308, 11.04)_=20.67, p=0.0005. Tukey Post-hoc, Weeks, 0/3, p=0.0237, 1/6, p=0.0014. **f.** Significant reduction in PCA trajectory lengths from week 0 to week 3, and week 0 to week 4 in susceptible mice. Susceptible mice (bottom): weeks, F_(1.483, 8.242)_=13.19, p=0.0039. Tukey Post-hoc, Weeks, 0/3, p=0.0012, 0/4, p=0.0192. All post-hoc comparisons are Tukey t-tests, *p<0.05, **p<0.01, ***p<0.001, ****p<0.0001.

We measured facial dynamics across weeks during stress and ketamine treatment in control, resilient, and susceptible groups by analyzing trajectory lengths post-event during reward trials (Extended Data Fig. 8a–c). Our results showed control mice exhibit a dramatic decrease following week 1 and remained consistent through most weeks (Extended Data Fig. 8a). Interestingly, in resilient mice, we observed dramatic peaks in facial trajectory lengths that began early stress (week 2), and continued late stress (week 5 and 6) and ketamine time points (Extended Data Fig. 8b). In stark contrast, susceptible mice revealed increased trajectory lengths during reward trials at late stress (Extended Data Fig. 8c). These data demonstrate distinct fluctuations in facial dynamics within stressed groups compared to control mice, supporting the notion that facial expression dynamics could provide a quantitative readout for diagnosis that would inform individualized treatment plans.

**Extended Data Figure 9.**
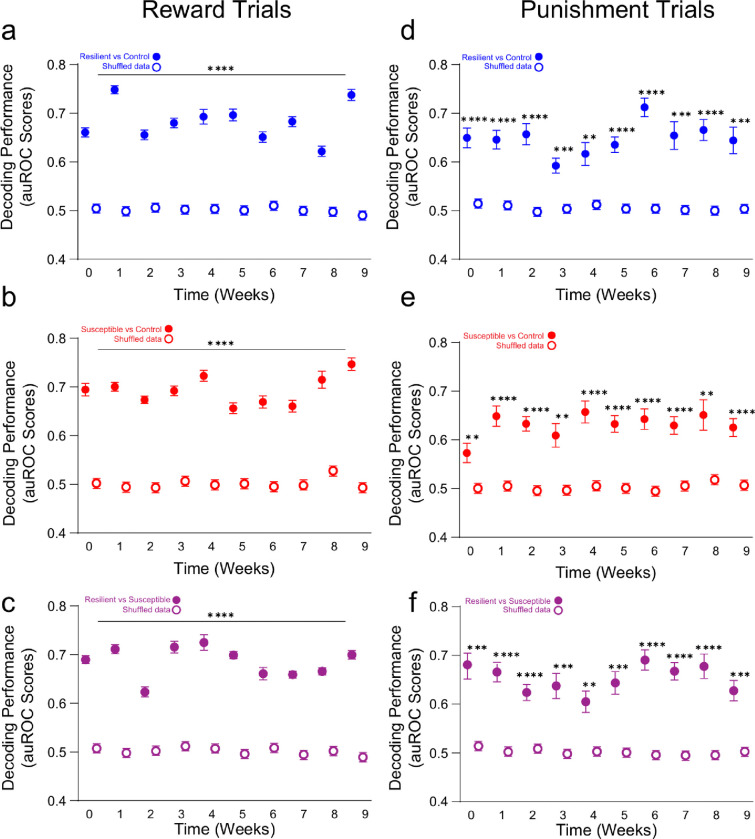
Facial dynamics are sufficient to decode stress phenotype across weeks and trial type. **a.** Significant decoding accuracy of *resilient vs control groups* compared to shuffled data within individual weeks. Decoding accuracy in response to sucrose in *resilient* vs *control* groups across weeks. Two-way Repeated Measures ANOVA, event F_(1,18)_=2222, p<0.0001, weeks F_(5.600, 100.8)_=9.127, p<0.0001, interaction F_(9,162)_=12.34, p<0.0001. Tukey Post-hoc, Weeks 1–9: p<0.0001. **b.** Significant decoding accuracy of *susceptible vs control groups* compared to shuffled data within individual weeks. Decoding accuracy in response to sucrose in susceptible vs *control* groups across weeks. Two-way Repeated Measures ANOVA, event F_(1,18)_=4044, p<0.0001, weeks F_(4.661,83.90)_=5.680, p=0.0002, interaction F_(9,162)_=4.642, p<0.0001. Tukey Post-hoc, Weeks 1–9: p<0.0001. **c.** Significant decoding accuracy of *resilient vs susceptible groups* compared to shuffled data within individual weeks. Decoding accuracy in response to sucrose in *resilient* vs *susceptible* groups across weeks. Two-way Repeated Measures ANOVA, event F_(1,18)_=5261, p<0.0001, weeks F_(5.097,91.75)_=8.356, p<0.0001, interaction F_(9,162)_=7.673, p<0.0001. Tukey Post-hoc, Weeks 1–9: p<0.0001. **d.** Significant decoding accuracy of *resilient vs control groups* compared to shuffled data within individual weeks. Decoding accuracy in response to Air puff in *resilient* vs *control* groups across weeks. Two-way Repeated Measures ANOVA, event F_(1,18)_=468.2, p<0.0001, weeks F_(5.367,96.61)_=1.764, p=0.1225, interaction F_(9,162)_=2.074, p=0.0347 **e.** Significant decoding accuracy of *susceptible vs control groups* compared to shuffled data within individual weeks. Decoding accuracy in response to Air puff in susceptible vs *control* groups across weeks. Two-way ANOVA, event F_(1,18)k_=238, p<0.0001, weeks F_(5.381,96.85)_=1.603, p=0.1618, interaction F_(9,162)_=1.108, p=0.3602. **f.** Significant decoding accuracy of *resilient vs susceptible groups* compared to shuffled data within individual weeks. Decoding accuracy in response to Air puff in *resilient* vs *susceptible* groups across weeks. Two-way Repeated Measures ANOVA, event F_(1,18)_=322.4, p<0.0001, weeks F_(5.184,93.31)_=1.463, p=0.2075, interaction F_(9,162)_=1.743, p=0.0831. All post-hoc comparisons are Tukey t-tests, ****p<0.0001 All 2-way ANOVAs were for event (event vs shuffle) and weeks (0–9).

To test whether we could predict if facial responses to reward stimuli could decode control, resilient and susceptible groups, we applied a generalized linear model (Fig. 4d). We showed efficient decoding performance of stress groups for sucrose trials over weeks (Extended Data Fig. 9a–c). Similar to neural decoding accuracy, we observed a significant increase in facial decoding performance in stress phenotypes compared to shuffled data at baseline, post-stress, ketamine, and post-ketamine time points (Fig. 4d, f, h, j). Interestingly, our results also showed a significantly higher decoding accuracy in susceptible vs *control* groups compared to *resilient* vs *control* groups after ketamine administration during reward trials (Fig. 4h).

Next, we examined facial dynamics across weeks in control, resilient and susceptible groups by measuring trajectory length difference score during punishment trials. Our results showed an increase in facial trajectory length difference score at baseline in resilient groups compared to control and susceptible mice (Fig. 4k). At post-stress time points, we observed no difference across groups in response to punishment stimuli (Fig. 4m). However, following ketamine administration, resilient mice displayed reduced facial dynamics compared to control mice (Fig. 4o). These results indicate that resilient mice exhibit significantly different facial responses to punishment stimuli during both baseline and ketamine treatment. Interestingly, during the post-ketamine time point, we noticed a significant increase in trajectory length difference score in susceptible mice compared to control and resilient groups (Fig. 4q).

We then measured facial dynamics across weeks during stress and ketamine treatment in control, resilient, and susceptible groups during punishment trials (Extended Data Fig. 8d–f). Our results showed an increase in trajectory lengths in control mice following ketamine administration (Extended Data Fig. 8d). Resilient subjects exhibit a reduction in trajectory length from week 0 to week 1, but increase in week 2 and week 6 time points (Extended Data Fig. 8e). In susceptible mice, we observed a decrease at early stress time points (week 3 and 4) during punishment trials (Extended Data Fig. 8f).

To test facial decoding performance in response to air puff between stress groups, we used a GLM and showed efficient decoding performance across weeks (Extended Data Fig. 9d–f). Next, we compared decoding accuracy between *resilient* vs *control* groups, *susceptible* vs *control* groups, and *resilient* vs *susceptible* groups during punishment trials at baseline, post-stress, ketamine, and post-ketamine time points (Fig. 4l, n, p, r). At baseline, we noticed an increase in *resilient vs*. *susceptible* decoding performance compared to *susceptible* vs *control* (Fig. 4l). However, we observed no difference among stress groups following post-stress and ketamine application (Fig. 4n, p, r). These results confirm that facial dynamics within groups are readily detectable during punishment stimuli, but are more discernable within resilient mice prior to stress.

## Conclusion

Together, these data revealed that mPFC valence-specific neural population activity and behavioral attributes predict anhedonia phenotypes. This study demonstrates that longitudinal tracking of neural populations and activity across epochs of unpredictable chronic mild stress can help identify biomarkers for depressive-like phenotypes. Stress subjects showed no difference in the reward/punishment ratio during late time points, whereas control mice displayed an increase in reward processing. Indeed, we demonstrate that mPFC neural dynamics and facial expression features can encode anhedonia at multiple time points. Susceptible mice displayed a significantly higher reward decoding accuracy compared to resilient mice at baseline, suggesting we can predict susceptibility prior to stress. Interestingly, chronic stress eliminates the neural decoding accuracy of punishment unconditioned stimuli in both resilient and susceptible groups.

We investigated the differential effects of ketamine application in both control and stressed groups, showing alleviation of anhedonia phenotypes within 24 hours that was sustained a week later. However, we demonstrate ketamine’s distinct stress-dependent changes during despair assays, where control mice show a reduction in mobility compared to both resilient and susceptible groups. Our data also highlights a preference in mPFC reward processing in stressed groups one week after ketamine administration. These data support the decoding studies, showing that susceptible mice exhibit higher decoding accuracy compared to resilient mice, which we speculate reflects an increased sensitivity to ketamine application within PFC dynamics and associated facial feature expressions. These data could lead to ketamine response predictions and sustainability, poised for subjects exposed to chronic stress. Altogether, this study highlights the importance of longitudinal data as a framework for identifying biomarkers of depressive-like phenotypes by analyzing granular behavioral attributes in combination with mPFC neural dynamic population features.

## Methods and Materials

### Animals and housing

Adult, male HET DAT-Cre genotyped mice (at the minimum age of 8 weeks) arrived from Jackson Laboratory (RRID: IMSRJAX:000,664) and bred at the Salk Institute, were utilized for this study. The mice were housed in a reverse light cycle, with ad libitum access to food and water, until the commencement of major survival surgery, behavioral tests or imaging sessions. The animals were accommodated in cages with up to three littermates mates. All animal handling procedures adhered to the guidelines stipulated by the National Institute of Health (NIH) and were approved by the UCSD Institutional Animal Care and Use Committee (IACUC).

### Stereotaxic surgery

Under aseptic conditions, surgery was conducted on all subjects using a small animal stereotax (David Kopf Instruments, Tujunga, CA, USA), with body temperature maintenance achieved using a heating pad. Anesthesia was induced using a 5% mixture of isoflurane and oxygen, which was subsequently reduced to 2–2.5% and maintained throughout the procedure (0.5 L/min oxygen flow rate). Once the subjects reached an adequate level of anesthesia, measured using a toe pinch, a 1mg/kg Buprenorphine-SR injection was administered subcutaneously, the ophthalmic ointment was applied to protect the eyes, hair was clipped from the incision site, the area was scrubbed alternatively three times with betadine and 70% ethanol, and lidocaine was subcutaneously (SQ) injected at the incision site. All measurements for viral injections were referenced from Bregma as the origin. Following the surgery, the subjects were IP injected with 1mL Ringer’s Lactate and placed in clean cages containing water-softened mouse chow to facilitate recovery. The cages were positioned on a heating pad to aid in the recovery process.

### Viral injection and GRIN lens placement surgery

To enable recordings from medial prefrontal cortex (mPFC) neurons, a viral approach was implemented. Following the aforementioned general surgical procedures, an incision was made to expose the skull. After skull leveling, craniotomies were performed above the mPFC regions. For expression of GCaMP, 300 nL of AAV1-hSyn-jGCaMP7f was injected into the mPFC at stereotaxic coordinates of 1.9 mm anteroposterior, 0.40 mm mediolateral, and −2.2 mm dorsoventral from Bregma. The injections were carried out using a 10 μL Nanofil syringe (WPI, Sarasota, FL, USA) driven at a rate of 0.1 μL/min with a microsyringe pump and controller (Micro4; WPI, Sarasota, FL, USA). Following each viral injection, the needle was allowed to stay in place for 5–10 minutes to allow viral material penetration before extraction. To prevent contamination, the needle was thoroughly flushed with 70% ethanol and sterile water. Viral aliquots were sourced from Addgene (Watertown, MA). Subsequent to viral injections, a 1 × 4 mm gradient refractive index (GRIN) lens (Proview, Inscopix Inc, Mountain View, CA, USA) was inserted into the mPFC at stereotaxic coordinates of 1.9 mm anteroposterior, 0.4 mm mediolateral, and −2.18 mm dorsoventral from Bregma. The GRIN lens was then secured to the skull and headplate using C&B Metabond and cement (Parkell), respectively.

## Behavioral testing

All behavioral testing occurred after a minimum of three weeks post-surgery recovery. Mice were individually handled for 15 minutes each day for five days to gain familiarity with experimenters and reduce stress during experiments.

### Sucrose preference test

The sucrose preference test (SPT) was used to measure anhedonia and was conducted in operant chambers (Med Associates, Inc) placed within sound-attenuated cubicles. Each SPT session lasted for 60 minutes and involved the use of two electrical lickometers and a house light set at an intensity of 40 lux. The lickometers were connected to bottles containing either tap water or a 1% sucrose solution in tap water. The MedPC IV software (Med Associates, Inc) was utilized to detect and record each lick event. Sucrose preference was calculated as (sucrose lick / (sucrose lick + water lick)) × 100. No additional food sources were available within the operant chambers. To ensure variability, the bottle configuration was different in each of the six operant chambers used. This allowed for repeated measures experiments, enabling animals to be re-tested and re-establish learning during each session.

### 3-Chamber Sociability test

The 3-chamber sociability test was used to measure sociability and was performed in a clear rectangular plexiglass arena. Prior to each session, the subject mouse is habituated in the empty arena for 3 minutes. Subsequently, the mouse is taken out of the arena, and a novel male mouse is placed inside a barred cup on one side of the arena together with an empty barred cup on the opposite side. The subject mouse is placed in the arena for 7 minutes during which footage is taken with a digital video camera above the arena. Ethovision XT software (Noldus, Wageningen, Netherlands) was used to record the mice during sociability assay.

### Tail suspension test

The tail suspension test was used to measure behavioral despair. The tail of each mouse was placed between two strips of autoclave labeling tape. The end of one strip of tape was then secured to a horizontal bar 40 cm from the ground, ensuring that the animal could not make other contact or climb during the assay. Video recording was started 90 s from the time that the animal was inverted and taped. Mice will be inverted for 6 minutes. Time spent struggling was measured by OD-log and blind scoring each minute of video material after the testing was completed and was reported in seconds for each minute of the assay.

### Unpredictable Chronic Mild Stress protocol

To induce anhedonic symptoms, the chronic mild stress (CMS) protocol was implemented within a mouse model^11^. Mice in the CMS group were exposed to 2–3 stressors per day for 6 weeks that consisted of cage tilting, strobe light illumination, white noise, crowded housing, light/dark cycle manipulations, food deprivation, water deprivation, and damp bedding. CMS mice were exposed to ~3–4 hours per day besides the 12 hr light/dark cycle stressors. Stressors were imposed over all cages and randomized across all the days. Control mice were not exposed to stressors.

### Ketamine administration

After the 6-week chronic mild stress protocol, all mice were IP injected with saline (0.01–0.04 ml). The following week all mice were IP injected with ketamine (1 mg/kg, 0.01–0.04 ml) to alleviate anhedonia. Mice were allowed to recover at least 24 hours after injection before performing behavioral tasks or imaging experiments.

### Anhedonia Classification

Mice were classified following chronic mild stress using unsupervised k-means clustering method (k=3). Number of clusters were determined by using the optimal k elbow method within-clusters sum of squares (WCSS). Groups were classified into control (non-stressed), resilient (stressed), and susceptible (stressed) groups.

## *In Vivo* 2-Photon calcium imaging

### Pavlovian discrimination paradigm and trial structure

In this Pavlovian paradigm, a highly palatable 30% sucrose solution (200 ms) served as the rewarding unconditioned stimulus (US), while a mildly punishment air puff to the subject’s face (~ 10 psi, 100 ms) acted as the punishment US. Both the rewarding and punishment US were paired with a 5-second pure tone as the conditioned stimulus (CS), with the tone frequency set at 9 kHz for the rewarding CS and 2 kHz for the punishment CS. The reward trial started with the CS followed by a lick contingent reward US with a 2-second delay. After the CS ended, the US was vacuumed away from the spout. The punishment trials started with the CS followed by the punishment US with a 2-second delay. The reward and punishment catch trials both consisted of the respective CS with no US. The trials were separated by a 25–30 second inter-trial interval (ITI).

Subjects were first head-fix trained in a closed box for 20 reward trials with no lick-contingency and no US delay. Each box was equipped with a replica of the acquisition setup, without the microscope. This consisted of a head-fix clamp fixed above the tube with the subject. A spout connected to a voltage recorder was fixed in front of the subject. The air-puff spout and camera were fixed to opposite sides of the subject. Training sessions were ramped up to 60 trials over 3 sessions, after which lick contingency was turned on with a 2-second US delay for 2 sessions. Subsequently, Discrimination training sessions started, where 20% of trials changed to punishment trials. Before acquisition trials started subjects were trained under the 2-photon microscope for another 3 sessions. If subjects did not perform correctly anticipatory lick responses to > 50% of reward trials, learning was deemed unsuccessful.

The acquisition sessions consisted of 8 punishment trials, 2 punishment catch trials (CS and no US), 36 reward trials without lick contingency, and 2 reward catch trials. These trials were pseudorandomized across the two blocks, with the requirements that the first 3 trials were reward trials, there were no consecutive sequences of 3 punishment trials, and the catch trials occurred in the last 15% of the trials. During each trial, facial footage, in vivo calcium imaging, and lick behavior was recorded.

### In vivo 2-photon calcium imaging

We used a two-photon microscope (Bruker Ultima Investigator, Bruker Nano) with a 20 × objective (0.45 NA, Olympus) and 920 nm excitation wavelength (Ti-Sapphire laser, Newport) for in vivo calcium imaging. Images were acquired using Prairieview (Bruker Nano) in resonant-galvo acquisition mode. Each field-of-view (FOV) (512 × 512 pixels covering 524 × 524 μm) was scanned at ~29.8 Hz.

### Signal processing

Images from 2-photon calcium imaging were processed using *Suite2P.* We used *Suite2P* to correct motion artifacts, define regions of interest (ROIs) corresponding to individual neurons, and extract their GCaMP fluorescence^29^. We selected only cellular ROIs by manual curation. Sessions and trials that contained motion artifacts and technical issues were taken out for further analysis. ROI match MATLAB software was used to identify cells that were successfully tracked across imaging sessions.

### Perfusion

Following the conclusion of recording experiments subjects were deeply anesthetized with an injection of sodium pentobarbital (200 mg/kg, intraperitoneal injection) and perfused transcardially with 20 mL of ice-cold lactated Ringer’s solution, followed by 20 mL ice-cold paraformaldehyde (4%; PFA) in phosphate-buffered saline (PBS). Brains were extracted and placed in 4% PFA for 24 h. The tissue was then equilibrated in a cryo-protectant solution (30% sucrose in PBS, w/v). Coronal slices measuring 60um were taken from the tissue using a sliding microtome (HM430; Thermo Fisher Scientific, Waltham, MA), and stored in PBS at 4 °C.

### Epifluorescence imaging

Tissue slices were imaged using an epifluorescence microscope (Keyence BZ-X). Images were taken using a 2x objective lens. Following imaging, the images were evaluated to determine the location of viral expression as seen via GCaMP7f. Recording sites were located using GRIN lens lesion locations.

### Principal component analysis

Principal component analysis (PCA) was used to measure population firing rate dynamics in the mPFC^30^. A local and global PCA was done on a matrix containing all Z-scored normalized data (Reward CS tone, Punishment CS tone, Reward first lick, Reward US, Punishment US) for all animals such that we could compare neural trajectories across groups (Control, Resilient, and Susceptible). For the local PCA, the matrix had neurons in rows, and in the columns had mean Z-score response during −10 to 10 seconds post CS event using 100 ms bins. The neural trajectories for each task-relevant event were created per group by multiplying the coefficients obtained in the PCA by the mean Z-score response across trials per week. For each neural trajectory, the length was calculated as the sum of Euclidean distances between adjacent 100 ms bins. Also, neural trajectories distances were calculated as the Euclidean distance between the two trajectories bin-by-bin. For statistical comparison analysis, the neural trajectory metrics were calculated using the leave-one-out (LOO) method, leaving out all the neurons from a single animal per group, therefore the number of iterations is the number of mice in that group. Thus, in every iteration the same PCA coefficients per cell were used for neural trajectory analysis. For quantification of trajectory lengths and distance between trajectories the first 23 PCs were used to capture 59.51% of the variance. For all trajectory visualizations and trajectory quantifications, we matched the number of neurons for each group (Control, Resilient, and Susceptible) for comparison analysis across weeks.

### Generalized linear model classifier

To test if anhedonia phenotype groups (Control, Resilient, and Susceptible) could be decoded during reward and punishment trials from mPFC population activity, we used a generalized linear model (GLM) classifier. To obtain anhedonia group mPFC population activity we used the coefficients obtained for each neuron in the local PCA and created a neural trajectory using the mean Z-score responses for the Reward and Punishment trials (Reward first lick and Punishment US). We trained the GLM using the first 8 PCs per session per week (−10 to 10 seconds post CS event) as features. We did a 10-fold cross-validation (CV), where the data was split into 10 subsets and in each iteration the training consisted of a different 90% subset of the data, then the testing was done with the remaining 10% of the data. For the 10-fold CV, we computed the area under the receiver operating characteristic curve (AUC score) for the test data. We used this model decode control versus resilient, control versus susceptible, and resilient versus susceptible. We then compared decoding performance (auROC scores) against shuffled data across weeks.

### SLEAP automated pose tracking analysis

#### Social analysis

To automatically detect social interaction behaviors, SLEAP^31^ was used to estimate animal poses in behavior recordings. We recorded behavior videos using Noldus EthoVision XT and a Basler GenI Cam at 25 frames/second, set at a fixed distance above the three-chamber arena. A training data set was labeled using a 12-point skeleton to represent the mouse (nose, head, neck, left ear, right ear, left forepaw, right forepaw, left hindpaw, right hindpaw, trunk, tail base, tail tip), and was used to train a bottom-up model consisting of 2399 frames. To define interaction behavior with the social and nonsocial cups, we used a distance threshold of within 1.3x pixels to the radius of the cup and an angle threshold of 90 degrees between the subject’s nose, body, and the center of each cup to quantify time spent interacting across frames.

#### Facial analysis

Video recordings of mouse facial expressions were collected on headfixed mice during discrimination sessions. We used SLEAP^32^ version 1.2.9 (https://github.com/talmolab/sleap) to estimate the position of facial keypoints using a 13-point custom facial skeleton. This consisted of 4 points for eye (upper_eye, lower_eye, inner_eye, outer_eye,), 2 for whiskers (top_whisker_stem, bottom_whisker_stem), 4 for nose (chin nose_upper, nose_tip, nostril_left, nostril_right), and 3 for mouth area (mouth_upper, mouth_lower, chin). Our SLEAP model consisted of a single-instance model with UNet backbone.

Analysis and visualizations were executed using MATLAB. We applied a smoothing filter to the SLEAP predictions using a Savitzky-Golay filter over a 5-frame window to minimize noise error associated with tracking. Using a custom-built MATLAB toolbox called Facial Expression Feature Extractor (FEFE), we extracted various facial features such as distances between keypoints, angles, velocities and accelerations of the nose and eye regions, and the areas of different facial regions. To reduce the bias of camera placement on our distance based features, we converted from pixels to cm by measuring the sucrose spout in each video and computing a pixel to cm conversion factor for that video.

We performed principal component analysis (PCA) on the total feature set across all sessions, normalizing each trial to a 5 s Baseline window immediately preceding that trial. To display PCA, we performed a leave-one-out analysis and averaged across results. To compute trajectory lengths, we computed the Euclidean norm of each subject’s trajectory, then took the mean across subjects. For distance between trajectories, we took the Euclidean norm of the pointwise differences of sucrose and airpuff trajectories for each time step for each session; from this we also computed average distance by phenotype.

For facial decoding, we projected the data into PCA space, then applied a multinomial logistic regression model. We used a 10-fold cross validation and compared the results to a control model where the phenotype labels were shuffled in random order. The area under the curve (AUC) metric was smoothed by applying a Gaussian moving average in a window using the previous 20 sec.

### Statistical methods

The thresholds for significance were placed at *p<0.05, **p<0.01, ***p<0.001, and ****p<0.0001 unless stated otherwise. All data are shown as mean and SEM. Wilcoxon signed rank-sum test, Pearson correlation, one-way ANOVA, Repeated-measure ANOVA, and mixed-effects model followed by a Tukey’s posthoc test were performed using GraphPad Prism 6 or MATLAB. The p values were corrected for multiple comparisons. Ward’s linkage hierarchical clustering utilizing Euclidean distance was performed using MATLAB.

## Extended Data

## Data Availability

All experimental data are available in the main text or supplementary material. Data will be available on an open-source database upon publication.
